# Dual inhibition of PRMT1 and SUV39H1 suppresses breast cancer progression and enhances immunotherapy response

**DOI:** 10.7150/ijbs.130955

**Published:** 2026-06-04

**Authors:** Ge Wang, Jiagui Song, Xinwei Duan, Kunhao Zhou, Jing Zhang, Hongquan Zhang, Yu Yu

**Affiliations:** 1 Department of Human Anatomy, Histology and Embryology, School of Basic Medical Sciences, PKU International Cancer Institute, Peking University Health Science Center, Beijing 100191, China.; 2 Center of Basic Medical Research, Institute of Medical Innovation and Research, Peking University Third Hospital, Beijing 100191, China.

**Keywords:** breast cancer, epigenetic inhibitor, PRMT1, SUV39H1, immunotherapy

## Abstract

Protein arginine methyltransferase 1 (PRMT1) dysregulation is frequently observed in various human cancers, including breast cancer. However, the antitumor efficacy of PRMT1 inhibitors remains limited in the treatment of breast cancer. Here, we propose a dual epigenetic inhibition strategy that effectively suppresses breast cancer growth and metastasis. We demonstrate that GSK3368715, a small-molecule inhibitor of PRMT1, downregulates the protein levels of the histone lysine methyltransferase SUV39H1 by enhancing its ubiquitination. Dual inhibition of PRMT1 and SUV39H1 results in significantly greater suppression of tumor growth and metastasis compared to either monotherapy, supporting the synergistic effects of targeting two epigenetic regulators. Consistently, dual inhibition markedly suppresses the growth of breast cancer organoids relative to single-agent treatments. Mechanistically, co-inhibition of SUV39H1 and PRMT1 enhances chromatin accessibility in promoter regions, thereby promoting the expression of key regulators involved in cell growth and migration. Furthermore, dual inhibition increases infiltration of CD8^+^ T cells and NK cells and upregulates PD-L1 expression. Importantly, the combination of dual inhibition with anti-PD-L1 antibody enhances the responsiveness of breast cancer to immunotherapy. Taken together, our findings indicate that co-targeting PRMT1 and SUV39H1 represents a promising therapeutic strategy for breast cancer.

## Introduction

Protein arginine methyltransferases (PRMTs) mediate arginine methylation in both histone and non-histone proteins, regulating a wide range of biological processes, including translation, RNA metabolism, DNA damage response, and stem cell maintenance 1-6. PRMTs are frequently dysregulated in various human cancers 7. Specific PRMT inhibitors have emerged as promising therapeutic agents for cancer treatment 8, 9, and their combination with other therapeutic modalities may offer clinical benefits to patients 10, 11. PRMT1 is the predominant type I PRMT responsible for catalyzing asymmetric dimethylarginine (ADMA) 12 and accounts for the majority of PRMT activity in mammalian cells. Previous studies have reported that PRMT1 mediates ADMA modification of certain histone methyltransferases, thereby enhancing their protein stability 13. PRMT1 inhibitors exhibit synergistic effects in suppressing tumor growth when combined with other small-molecule inhibitors 14, 15. Pharmacological inhibition of PRMT1 in combination with chemotherapy gemcitabine has a synergistic effect on pancreatic tumor growth 16. However, PRMT1 inhibitors alone exert minimal inhibition on tumor growth. Therefore, combination therapy represents the most effective strategy to overcome this limitation.

Suppressors of variegation 3-9 homologs (SUV39H) are specific H3K9 methyltransferases 17. SUV39H1-dependent trimethylation of histone H3 lysine 9 (H3K9me3) is essential for heterochromatin maintenance and genome stability 18, 19. SUV39H1 inhibition activates antiviral pathways and enhances cancer cell immunogenicity 20. SUV39H1 can function as either a tumor suppressor or an oncogenic promoter, depending on the cancer type. SUV39H1 is downregulated in various leukemias, and its knockdown accelerates disease progression by expanding the leukemia stem cell population 21. In contrast, SUV39H1 is frequently upregulated in solid tumors, where it promotes mammary tumor progression and has been identified as a potential biomarker for hepatocellular carcinoma diagnosis 22, 23. A recent study reports that ASB7 acts as an E3 ubiquitin ligase targeting SUV39H1, rendering cancer cells more susceptible to poly (ADP-ribose) polymerase inhibitors 24. In addition, inhibition of SUV39H1 activates cGAS-STING signaling, and targeting the SUV39H1-cGAS axis can promote the efficacy of radiotherapy 25. However, SUV39H1 inhibitors fail to sufficiently suppress tumor growth when used as monotherapy. Therefore, we propose that combining SUV39H1 inhibitors with other epigenetic inhibitors or immune checkpoint blockade represents a promising therapeutic strategy for cancer treatment.

In this study, we aim to evaluate the combined effect of two epigenetic inhibitors on breast cancer progression. Our results demonstrate that dual inhibition of PRMT1 and SUV39H1 significantly suppresses breast tumor growth and metastasis both *in vitro* and *in vivo*. Notably, the integration of immune checkpoint blockade with this dual-inhibitor regimen produces a synergistic antitumor response. Collectively, co-targeting PRMT1 and SUV39H1 represents a promising strategy for the treatment of breast cancer.

## Materials and Methods

### Cell culture

Human breast cancer cells lines were obtained from the Cell Resource Center, Peking Union Medical College (the Headquarters of National Infrastructure of Cell Line Resource, NSTI). Human breast cancer cell lines were cultured in DMEM supplemented with 10% FBS (Invitrogen, Carlsbad, CA, USA), 100 units/mL penicillin and 0.1 mg/mL streptomycin at 37℃ under 5% CO2 in a humidified incubator.

### Western blot analysis and co-immunoprecipitation (Co-IP)

Western blot and Co-IP assays were performed as described previously 26. We used anti-SUV39H1 (active motif, 39785), anti-PRMT1 (abcam, ab190892), anti-GAPDH (abclonal, AC002), anti-HA (abcam, ab9110), and anti-Flag (Sigma-Aldrich, F1804).

### Cell proliferation and colony formation

Cells were plated into 96-well plates at 1000 cells/well. WST-1 (Roche) (10 μl/well) was added into the cells and incubated at 37℃ for 2 h. The absorbance at 450 nm was then measured in a microplate reader. For colony formation, 1000 cells were plated into 6-well plates. Two weeks later, cells were fixed, stained with crystal violet, and photographed.

### TAXIScan-FL assay

SUM159 cells were dissociated into single cells and the concentration was adjusted to 1×10⁶ cells/mL. The TAXIScan-FL cell dynamic analysis system was used to continuously record cell motility trajectories over a period of 5-6 hours, followed by analysis of migration distance and motility speed.

### Animal models

BALB/c nude mice and C57BL/6N mice (4-6 weeks old) were purchased from the Animal Experimental Center of Peking University Health Science Center and raised in a specific pathogen-free animal laboratory. The Ethics Committee of Peking University Health Science Center approved the mouse experiments (Permit Number: LA2020183) for this study. The mice were handled in accordance with the ethical standards of the Helsinki Declaration of 1975 and the revised version in 1983.

Xenografts in mice: MDA-MB-231 cells were inoculated orthotopically onto the abdominal mammary fat pad of nude mice (5×10^6^ cells/mouse), and py8119 cells were inoculated orthotopically onto the abdominal mammary fat pad of C57BL/6N mice (4×10^5^ cells/mouse). Tumor volume (V=length×width×width×0.5mm^3^) was measured at regular intervals. After the mice were sacrificed, the tumors were weighed and photographed. Left cardiac ventricle injection model: Py8119-luci cells were inoculated into the left cardiac ventricle of C57BL/6N mice (1×10^5^ cells /mouse) through VisualSonics Vevo3100 (FUJIFILM). After 2-4 weeks, D-luciferin potassium salt was injected into the peritoneal cavity of each mouse, the luciferase signal was detected by IVIS Spectrum (PerkinElmer).

### Reagents

GSK3368715 (MCE, Cat#HY-128717A) (100-150 mg/kg, once every two days), chaetocin (MCE, Cat# HY-N2019) (0.5-0.75 mg/kg, once every two days), and F5446 (MCE, Cat#HY-150190) (20mg/kg, once every two days) were administered alternately by intraperitoneal injection. InVivoMAb anti-mouse PD-L1 (BioXCell, Cat#BE0101) (300 μg/mouse, once every four days) was administered by intraperitoneal injection.

### Flow cytometry

Tumors from mice were dissociated into single cells and filtered by a 70 mm filter. Cell surface staining was performed with the indicated antibodies: BV605 anti-CD45 (Biolegend, 103140), FITC anti-CD3 (Biolegend, 100203), PE/Cyanine7 anti-CD8a (Biolegend, 100722), PE anti-NK1.1 (Biolegend, 156504), FITC anti-F4/80 (Biolegend, 123107), and PE anti-CD11b (Biolegend, 101207) antibodies. All antibodies were purchased from BioLegend. Samples were analyzed using a flow cytometer and data analyzed with FlowJo Software.

### RNA isolation and quantitative real-time PCR (qPCR)

Total RNA was isolated from tumor tissues with TRIzol reagent (Invitrogen) according to the manufacturer's instructions. cDNA was synthesized using a HiScript II Q RT SuperMix Kit (Vazyme). Real-time PCR was performed using a ChamQ SYBR qPCR Master Mix (Vazyme) by LightCycler 96 detection system (Roche). Mouse qPCR primers:

cGAS-F-CAGGAAGGAACCGGACAAGC, R-CCGACTCCCGTTTCTGCATT;

STING-F-GGTCACCGCTCCAAATATGTAG, R-CAGTAGTCCAAGTTCGTGCGA;

RIG-I-F-CAGATCCGAGACACTAAAGGGA, R-TCCTCATCAGCCTTGCTTTCA;

MDA5-F-ATGGACGCAGATGTTCGTGG, R-TCCCTTCTCGAAGCAAGTGTC;

MAVS-F-CTGCCTCACAGCTAGTGACC, R-CCGGCGCTGGAGATTATTG;

IFNβ-F-GGTGGAATGAGACTATTGTTG, R-AGGACATCTCCCACGTC;

CXCL9-F-TGAGGTCTTTGAGGGATTTGTAGTG, R-GGAACCCTAGTGATAAGGAATGCA;

CXCL10-F-GACGGTCCGCTGCAACTG, R-CTTCCCTATGGCCCTCATTCT;

PD-L1-F-GACCAGGTTTTGAAGGGAAATG, R-CTGGTTGATTTTGCGGTATGG;

GAPDH-F-TGACCTCAACTACATGGTCTACA, R-CTTCCCATTCTCGGCCTTG.

### ATAC-seq

FASTQ files were checked with FastQC. Raw reads were trimmed with Fastp and aligned to the hg38 genome using Bowtie2. BAM files were sorted and indexed with SAMtools. Peaks were called using MACS2 in narrow peak mode. BigWig files were generated with deepTools bamCoverage. Heatmaps and profile plots were created using deepTools. Peaks were annotated with ChIPseeker. GO and KEGG enrichment analyses were performed and visualized with ClusterProfiler.

### RNA-seq

FASTQ files were checked with FastQC. Raw reads were trimmed with Fastp and aligned to the hg38 genome using HISAT2. Gene expression was quantified from BAM files using FeatureCounts. Differentially expressed genes were identified with EdgeR. GO and KEGG enrichment analyses were performed and visualized using ClusterProfiler. GSEA was done with enrichplot.

### Tumor specimens and immunohistochemistry staining

Human breast cancer tissue microarrays were purchased from the National Human Genetic Resources Sharing Service Platform2005DKA21300 (Shanghai Outdo Biotechnology Company Ltd., China). The Ethics Committee of Shanghai Outdo Biotechnology Company approved the study (Permit Number: SHYJS-CP-1804004). Tissue sections were subjected to immunohistochemical staining using anti-PRMT1 and anti-SUV39H1 antibodies. Staining intensity was semi-quantitatively scored on staining level: 0 (negative), 1+ (weak), 2+ (moderate), and 3+ (strong). Based on the median immunostaining score for each protein, samples were divided into high-expression and low-expression groups. Survival curves were estimated by the Kaplan-Meier method, and survival rates in different groups were compared by the log rank test.

Tissue sections were deparaffinized and gradually rehydrated. Endogenous peroxidase activity was blocked by incubation with 3% hydrogen peroxide for 30 minutes at room temperature, followed by antigen retrieval in sodium citrate buffer (pH 6.0) for 20 minutes in a 100 °C water bath. The sections were then incubated with primary antibodies overnight at 4 °C. Detection was performed using the PV9000 2-step plus Poly-HRP anti-mouse/rabbit IgG (Zhong Shan Jin Qiao). Diaminobenzidine (DAB) was used as the chromogen (ChemMate Detection Kit, DAKO, Glostrup, Denmark), and hematoxylin served as the counterstain.

### Organoid preparation and culture

Patient-derived breast cancer organoids were generated from fresh or cryopreserved tumor tissues. Tissue samples were minced and digested using a dissociation reagent (Beijing Daxiang Technology Co., Ltd.). The cell suspension was centrifuged, filtered through a 70 μm strainer, and mixed 1:1 with growth factor reduced Matrigel (Corning). After seeding as 30-50 μL droplets and polymerizing at 37 °C for 30 min, Matrigel domes were overlaid with specialized breast cancer organoid medium (Beijing Daxiang Technology Co., Ltd.). Organoids were cultured at 37 °C with 5% CO₂, with medium refreshed every 2-3 days and passaged every 7-14 days via mechanical/enzymatic dissociation.

### Organoid viability assay

Organoids were dissociated, embedded in Matrigel, and seeded into 96-well plates. After a 3-day recovery period, organoids were treated for 72 h with the indicated agents: 0.1% DMSO (vehicle control), 200 nM GSK, 100 nM Chaetocin, the combination of GSK and Chaetocin. To visualize live and dead cells, organoids were stained with AO/PI and incubated at 37 °C for 1 h, followed by imaging using a high-content confocal microscope (Perkin Elmer CLS Operetta). Viability was quantified with the CellTiter-Glo 3D Cell Viability Assay (Promega) using a GloMax Luminometer (Promega).

### LC-MS/MS analysis

Total proteins were extracted from MCF7 cells treated with either DMSO (vehicle control) or GSK3368715 (2 μM, 48 h) using RIPA lysis buffer, followed by liquid chromatography-tandem mass spectrometry (LC-MS/MS) analysis. Label-free quantification (LFQ) was performed to determine relative protein abundances. Raw MS data were processed using MaxQuant (v2.0.3.0) against the UniProt Human reference proteome, with peptide-spectrum match (PSM) and protein-level false discovery rates (FDR) both set to ≤ 1%. Protein abundance was calculated based on the integrated signal intensity of peptide peaks. Proteins exhibiting a fold change ≥ 2.0 or ≤ 0.5 and an adjusted P value < 0.05 were classified as differentially expressed based on data from four independent experiments.

### Statistical analysis

All statistical analyses were performed using GraphPad Prism software. Unpaired t-tests with Welch's correction were used to compare two groups, two-tailed, 95% CI. One-way ANOVA combined with Tukey's multiple comparisons test was employed for analyses involving more than two groups. Growth curves were analyzed using two-way ANOVA with Geisser-Greenhouse correction. All results are presented as mean ± SD. Statistical significance was defined as P < 0.05, with a 95% confidence interval.

## Results

### Dual inhibition of PRMT1 and SUV39H1 effectively suppresses breast cancer growth and metastasis

**Figure 1:**
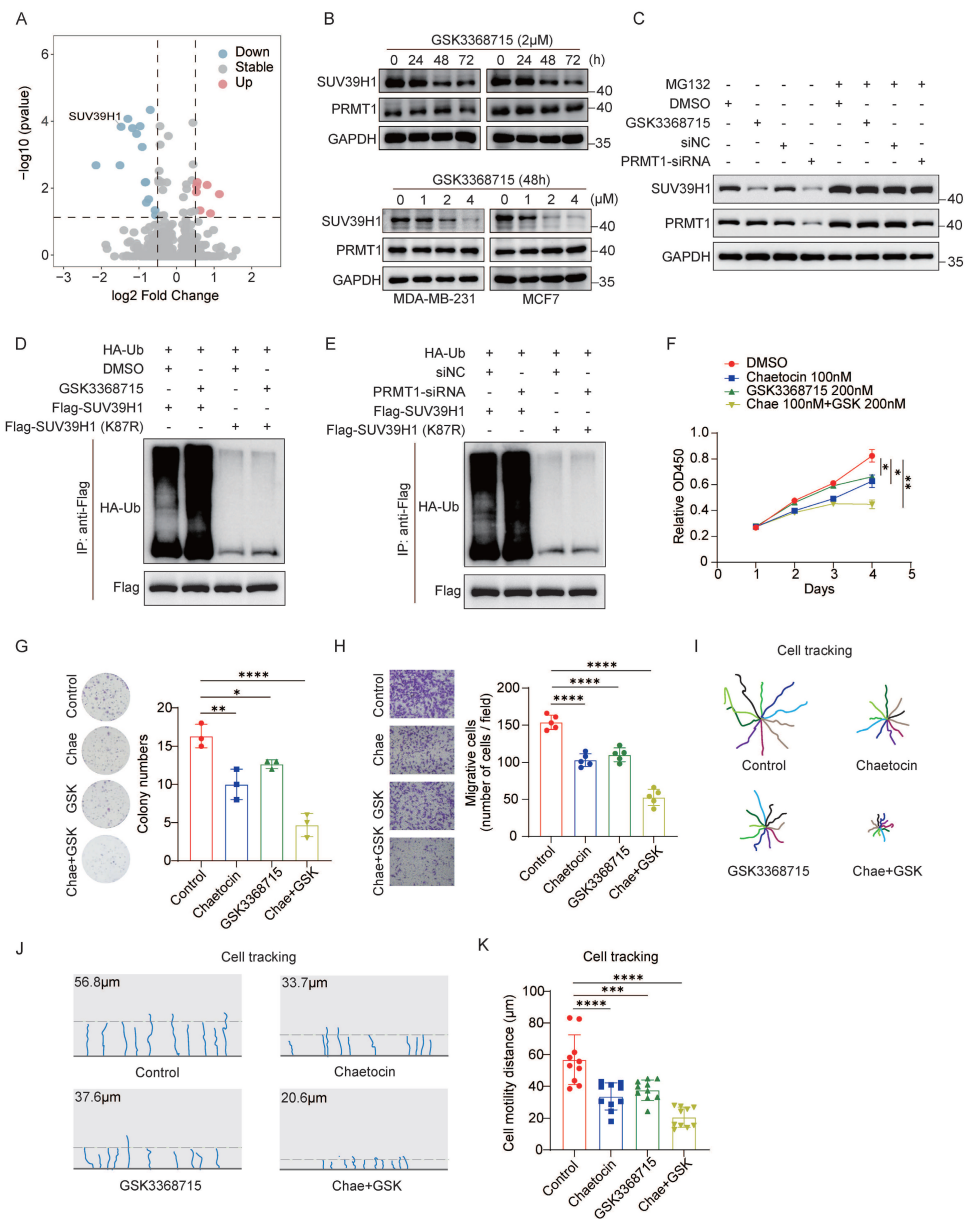
Dual inhibition of PRMT1 and SUV39H1 suppresses breast cancer cell proliferation and migration *in vitro*. (A) Total proteins were extracted from MCF7 cells treated with the PRMT1 inhibitor GSK3368715 (2 μM, 48 h) for LC-MS/MS. Volcano plots showing differentially expressed proteins in GSK3368715-treated versus untreated cells (P < 0.05; fold change ≥ 2.0 or ≤ 0.5) from four independent experiments. (B) Both MDA-MB-231 and MCF7 cells were treated with GSK3368715 at different time points and various concentrations, followed by Western blotting. (C) MDA-MB-231 cells were treated with GSK3368715 (2μM, 48 h) or PRMT1 siRNA (48 h), together with MG132 (25μM, 6 h), followed by Western blotting. (D) HA-ub and Flag-SUV39H1 (WT or K87R) was transfected into MDA-MB-231cells. Cells were treated with GSK3368715 (2 μM, 48 h) and MG132 (25μM, 6 h). Flag-SUV39H1 ubiquitination was detected by Co-IP. (E) HA-ub and Flag-SUV39H1 (WT or K87R) was transfected into control or PRMT1 siRNA-treated MDA-MB-231cells. Flag-SUV39H1 ubiquitination was detected by Co-IP. (F) MDA-MB-231 cells were treated by GSK3368715 (200 nM) and/or chaetocin (100 nM) followed by WST-1 assays. *p< 0.05, **p< 0.01 by two-way ANOVA with Geisser-Greenhouse correction. (G-H) MDA-MB-231 cells were treated with GSK3368715 (200 nM) and/or chaetocin (100 nM), and followed by colony formation assays and transwell assays. (I-K) MDA-MB-231 cells were treated with GSK3368715 (200 nM) and/or chaetocin (100 nM), followed by TAXIScan-FL. The movement trajectories of individual cells (I-J), and the distance of cell motility for each group (K) were analyzed. Data represent the mean ±SD, *p < 0.05, **p < 0.01, ***p < 0.001, ****p < 0.0001 by one-way ANOVA with multiple comparisons (G, H, K).

GSK3368715, a well-characterized inhibitor of PRMT1, represents a promising therapeutic candidate for cancer treatment. To explore novel combination strategies involving GSK3368715, we performed mass spectrometry (MS) analysis in MCF7 cells treated with GSK3368715. Gene Ontology analysis revealed that the differentially regulated proteins were predominantly enriched in biological processes including negative regulation of cell cycle and histone modifying activity (Supplementary Fig. 1A). Notably, the protein level of histone lysine methyltransferase SUV39H1 was markedly decreased following GSK3368715 treatment (Fig. 1A). Thus, we first investigated the effect of GSK3368715 on SUV39H1 protein levels. GSK3368715 treatment markedly reduced SUV39H1 protein level in a time- and dose-dependent manner (Fig. 1B). However, the mRNA levels of SUV39H1 remained unchanged (Supplementary Fig. 1B), suggesting that GSK3368715 regulates SUV39H1 degradation. Next, treatment with the proteasomal inhibitor MG132 reversed the downregulation of SUV39H1 protein levels induced by either GSK3368715 or PRMT1 siRNA (Fig. 1C). Furthermore, both pharmacological inhibition and genetic knockdown of PRMT1 significantly increased SUV39H1 ubiquitination; this effect was fully abrogated by the degradation-resistant SUV39H1 mutant (K87R) (Fig. 1D-E). These results demonstrate that PRMT1 inhibition by GSK3368715 triggers SUV39H1 ubiquitination and subsequent proteasomal degradation.

To explore a potential therapeutic strategy, we first evaluated the effects of PRMT1 and SUV39H1 inhibitors on breast cancer cell proliferation and migration. MDA-MB-231 cells were treated with the PRMT1 inhibitor GSK3368715 and the SUV39H1 inhibitor chaetocin, followed by WST-1 cell proliferation assays. The results showed that both GSK3368715 and chaetocin inhibited breast cancer cell proliferation in a dose-dependent manner (Supplementary Fig. 1C-F). Next, we confirmed a synergistic effect between GSK3368715 and chaetocin using the drug interaction model (HAS model) (Supplementary Fig. 1G). We selected pharmacologically relevant concentrations—200 nM GSK3368715 and 100 nM chaetocin—for combination treatment of MDA-MB-231 cells and assessed their impact on proliferation and migration. The combination of GSK3368715 and chaetocin significantly enhanced the suppression of cell proliferation compared to either agent alone (Fig. 1F). Additionally, dual inhibition markedly reduced colony formation compared to either inhibitor alone (Fig. 1G), an effect phenocopying that induced by PRMT1 or SUV39H1 knockdown (Supplementary Fig. 1H-I). Next, Transwell assays confirmed that the dual inhibition significantly impaired breast cancer cell migration (Fig. 1H). Moreover, we utilized the TAXIScan-FL assay to track and quantitatively assess cell migration and motility. Inhibition of either PRMT1 or SUV39H1 reduced the migration distance of breast cancer cells. Notably, the combination of the two inhibitors significantly suppressed migration distance to a greater extent than either inhibitor alone (Fig. 1I-K). These results demonstrate that the combined treatment with GSK3368715 and chaetocin exerts a synergistic effect in inhibiting breast cancer cell proliferation and migration *in vitro*.

**Figure 2:**
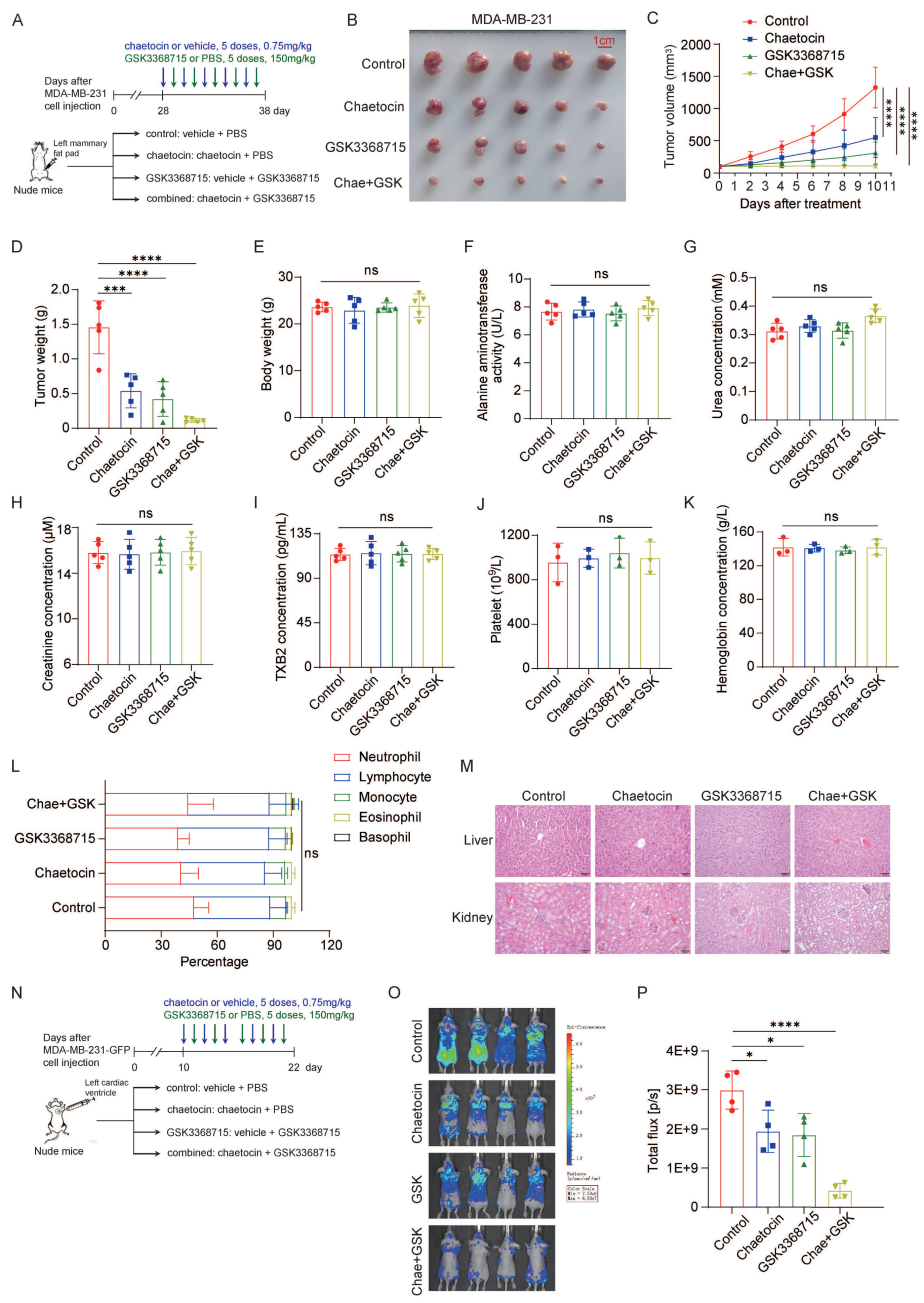
Combination therapy of PRMT1 and SUV39H1 inhibitors suppresses breast cancer growth and metastasis *in vivo*. (A-M) A schematic illustration of the experimental setup for MDA-MB-231 cells *in vivo* (A). MDA-MB-231 cells were inoculated onto mammary fat pad of nude mice. After 4 weeks, the mice were divided into 4 groups (n=5/group) and followed the intraperitoneal injection with inhibitors once every 2 days. Images of primary tumor (B), tumor growth curve (C), tumor weights (D), mice weights (E), blood ALT activity (F), BUN concentration (G), creatinine concentration (H), TBX2 concentration (I), platelet count (J), hemoglobin concentration (K), and white blood cell count (L). H&E staining of the liver and kidney tissues (M). (N-P) MDA-MB-231-GFP cells were injected into nude mice through heart injection. Mice were divided into 4 groups (n=4/group) and treated with the inhibitors by intraperitoneal injection every two days (N). IVIS imaging on the 22nd day (O), and the fluorescence intensity (P). Data represent the mean ±SD, *p < 0.05, **p < 0.01, ***p < 0.001, ****p < 0.0001 by two-way ANOVA with Geisser-Greenhouse correction (C) and one-way ANOVA with multiple comparisons (D-L, P).

Furthermore, we employed nude mice bearing xenograft tumors to evaluate the *in vivo* antitumor efficacy of chaetocin alone, GSK3368715 alone, or their combination (Fig. 2A). Tumor growth was monitored over time, and at the experimental endpoint, both tumor volume and final tumor weight were significantly reduced in the combination group compared to either monotherapy group (Fig. 2B-D). To evaluate the systemic toxicity of the dual-inhibitor regimen, we conducted a comprehensive assessment including monitoring of body weight, serum biochemical parameters, hematological indices, and histopathological examination of major organs. No statistically significant changes in body weight were observed among the four groups (Fig. 2E). Serum analysis revealed no significant alterations in alanine aminotransferase (ALT), urea nitrogen (BUN), creatinine, or thromboxane B2 (TXB2) levels (Fig. 2F-I). Similarly, hematological indices—including platelet count, hemoglobin concentration, and white blood cell count—showed no significant differences across all four experimental groups (Fig. 2J-L). Histopathological evaluation of the liver, kidneys, small intestine, and stomach revealed no structural abnormalities (Fig. 2M and Supplementary Fig. 2A). Collectively, these data indicate that the dual-inhibitor combination is well tolerated in this murine model.

As previously reported, loss of PRMT1 or SUV39H1 induces spontaneous DNA damage. To assess this effect, we detected the formation of γH2A.X foci, a well-established marker of DNA double-strand breaks. Data showed that either individually or in combination significantly increased the number of γH2A.X foci *in vivo* (Supplementary Fig. 2B), an effect phenocopying that induced by PRMT1 knockdown (Supplementary Fig. 2C), indicating that induction of DNA damage plays an important role in the antitumor activity of dual inhibition. Additionally, the expression level of Ki67 is significantly decreased upon treatment with dual inhibitors, confirming that dual inhibition of PRMT1 and SUV39H1 markedly suppresses tumor cell proliferation (Supplementary Fig. 2D).

Given the inhibitory effects of the two compounds on cancer cell migration *in vitro*, we next evaluated their efficacy in a metastatic mouse model. MDA-MB-231-Luc-D3H2LN cells were inoculated intracardially into nude mice, and treatment with chaetocin, GSK3368715, or their combination was initiated 10 days after inoculation (Fig. 2N). As expected, the combination group exhibited lower metastatic signals compared to either chaetocin or GSK3368715 monotherapy groups (Fig. 2O-P). Collectively, these results demonstrate that combined inhibition of PRMT1 and SUV39H1 has therapeutic potential for suppressing breast cancer growth and metastasis.

### Combination therapy targeting PRMT1 and SUV39H1 enhances immunotherapy responses

**Figure 3:**
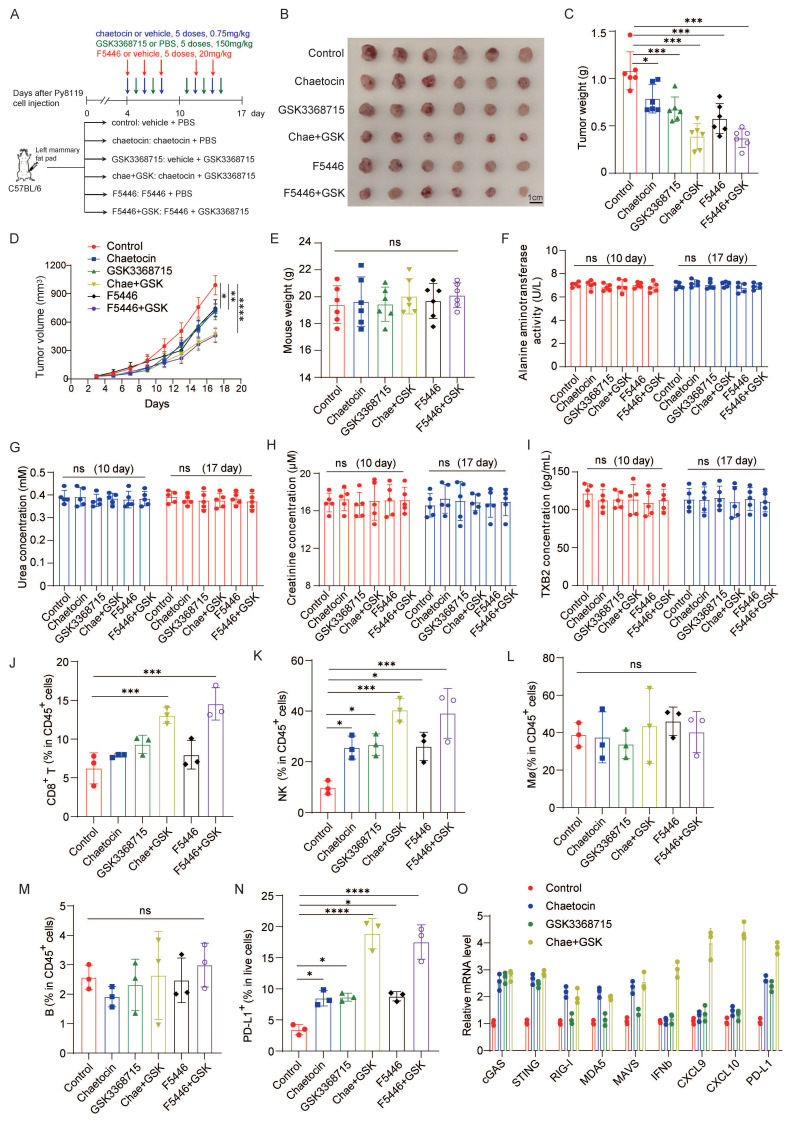
Combination therapy with PRMT1 and SUV39H1 inhibitors enhances immune cell infiltration. (A-I) A schematic illustration of the experimental setup for Py8119 cells *in vivo* (A). Mouse py8119 cells were inoculated onto mammary fat pad of C57 mice (n=6/group). Images of primary tumor (B), tumor weights (C), tumor growth curve (D), mice weights (E), blood ALT activity (F), BUN concentration (G), creatinine concentration (H), TBX2 concentration (I). (J-N) Tumors from six groups were digested into a single cell suspension for multiparametric flow cytometry. Cell populations of CD8^+^ T cells, NK cells, macrophages, B cells, and PD-L1^+^ cells were quantified. (O) Py8119 cells were treated with GSK3368715, chaetocin or the combination. The total RNA was extracted for RT-qPCR. Data represent the mean ±SD, *p < 0.05, **p < 0.01, ***p < 0.001, ****p < 0.0001 by two-way ANOVA with Geisser-Greenhouse correction (D) and one-way ANOVA with multiple comparisons (C, E-N).

Both SUV39H1 and PRMT1 inhibitors were administered to immunocompetent mice bearing tumors to evaluate their therapeutic potential in the context of an intact immune system. In addition to chaetocin, another selective SUV39H1 inhibitor F5446 also exhibited significant synergy with GSK3368715 (Supplementary Fig. 3A-C). We therefore treated C57BL/6 mice bearing Py8119 tumors with either the GSK3368715-chaetocin or the GSK3368715-F5446 combination regimen (Fig. 3A). Both tumor volume and weight were decreased in mice treated with either inhibitor alone compared to controls. Notably, the combination therapy resulted in a more pronounced reduction in tumor size and weight than either monotherapy, further supporting the synergistic effect of two epigenetic inhibitors (Fig. 3B-D). No statistically significant changes in body weight, blood ALT, BUN, creatinine, TXB2 levels, or hepatic and renal histological architecture were observed across all the experimental groups (Fig. 3E-I and Supplementary Fig. 3D), indicating that the dual-inhibitor combination is well tolerated in C57BL/6 mice model.

Moreover, we assessed intratumoral immune cell infiltration by multiparametric flow cytometry and found that combination therapy significantly increased the infiltration of CD8^+^ T cells and natural killer (NK) cells within tumors, whereas no significant changes were observed in macrophages or B cells (Fig. 3J-M and Supplementary Fig. 4A). Flow cytometry analysis also revealed a marked increase in the proportion of PD-L1-positive tumor cells following treatment with either chaetocin or GSK3368715 alone, and a further enhancement upon combination treatment (Fig. 3N and Supplementary Fig. 4B).

Given to the established link between PRMT1 or SUV39H1 inhibition and dsDNA or dsRNA sensing pathways, we quantified mRNA expression levels of key components in these pathways in py8119 cells treated with chaetocin, GSK3368715, or their combination by RT-PCR. We found that either PRMT1 or SUV39H1 inhibition activated the cGAS-STING pathway; however, only SUV39H1 inhibition induced activation of the RIG-I-MDA5-MAVS pathway. Notably, combination treatment synergistically upregulated IFN-β and IFN-stimulated genes including CXCL9 and CXCL10, as well as PD-L1. These findings demonstrate functional cooperativity between PRMT1 and SUV39H1 inhibition in activating IFN-β signaling (Fig. 3O).

**Figure 4:**
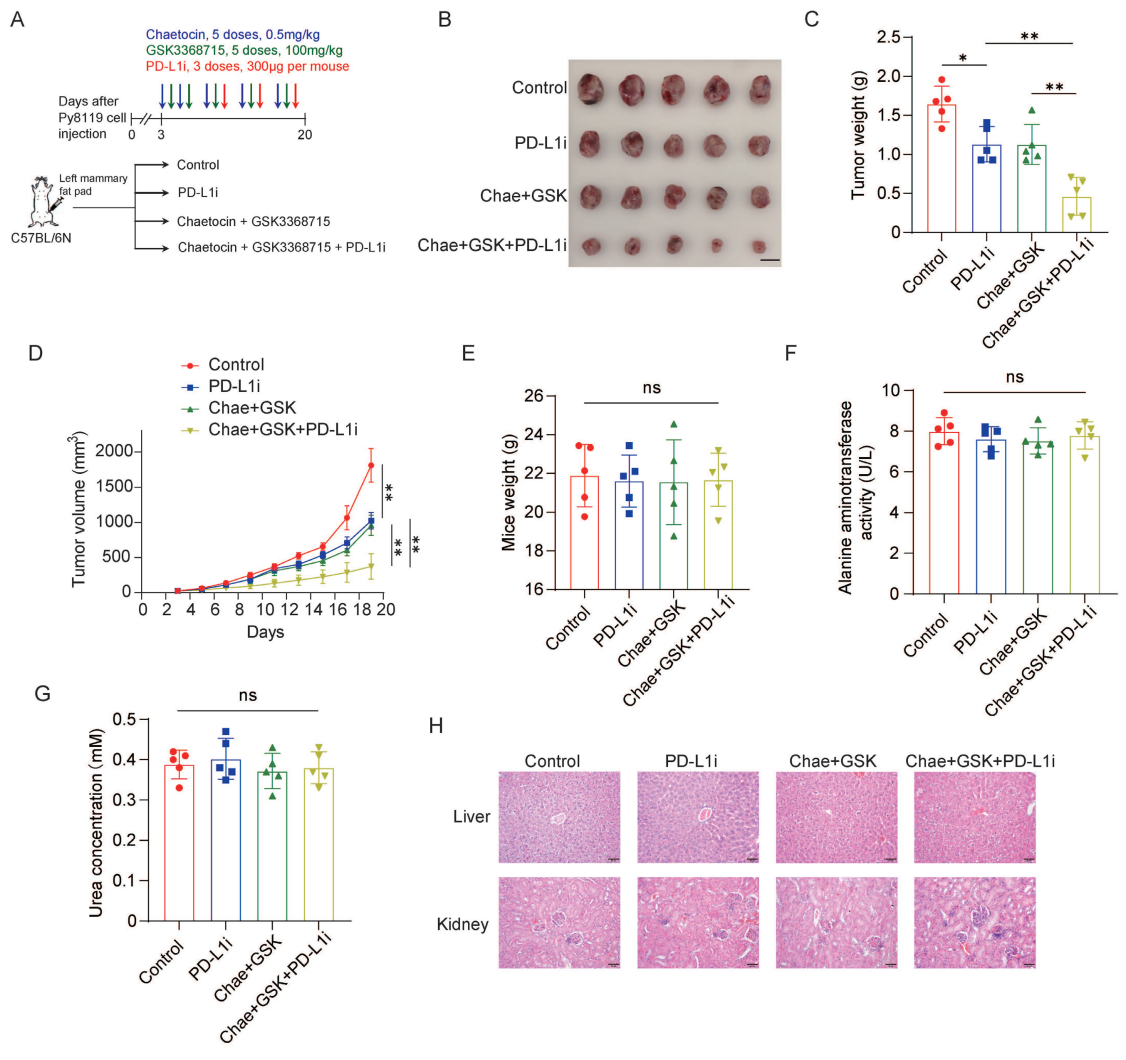
Dual inhibition enhances the response to immunotherapy. (A) Py8119 cells were injected into C57BL/6N mice. Mice were divided into 4 groups (n=5/group) and administrated with dual inhibitors and/or InVivoMAb anti-PD-L1. (B) Images of primary tumor. (C) Tumor weights. (D) Tumor growth curve. (E) Mice weights. (F) Blood ALT activity. (G) BUN concentration. (H) HE staining of the liver and kidney tissues. Data represent the mean ±SD, *p < 0.05, **p < 0.01, ***p < 0.001, ****p < 0.0001 by two-way ANOVA with Geisser-Greenhouse correction (D), and one-way ANOVA with multiple comparisons (C, E, F, G).

Next, we investigated whether dual inhibition of PRMT1 and SUV39H1 potentiates response to immune checkpoint blockade. Immunocompetent C57BL/6 mice bearing Py8119 syngeneic mammary tumors were treated with the dual-inhibitor regimen (GSK3368715 plus chaetocin), anti-PD-L1 antibody, or their combination. To enhance the therapeutic relevance of immunotherapy and reduce the potential off-target toxicity, the doses of both inhibitors were reduced by one third. The dual inhibitors were administered for two cycles; anti-PD-L1 was then initiated to establish the combinatorial immunomodulatory regimen (Fig. 4A). As expected, the triple-combination therapy—dual inhibitors plus anti-PD-L1—significantly suppressed tumor growth compared with either dual inhibition alone or anti-PD-L1 monotherapy, demonstrating a synergistic antitumor effect (Fig. 4B-D). No significant differences were observed among the four groups in body weight change (Fig. 4E), serum ALT activity (Fig. 4F), BUN concentration (Fig. 4G), or histopathological architecture of liver and kidney tissues (Fig. 4H), indicating that the combination regimen is well tolerated. Collectively, these findings demonstrate that dual pharmacological inhibition of PRMT1 and SUV39H1 synergizes with anti-PD-L1 therapy to enhance antitumor immunity.

### Dual inhibition increases the chromatin accessibility

**Figure 5:**
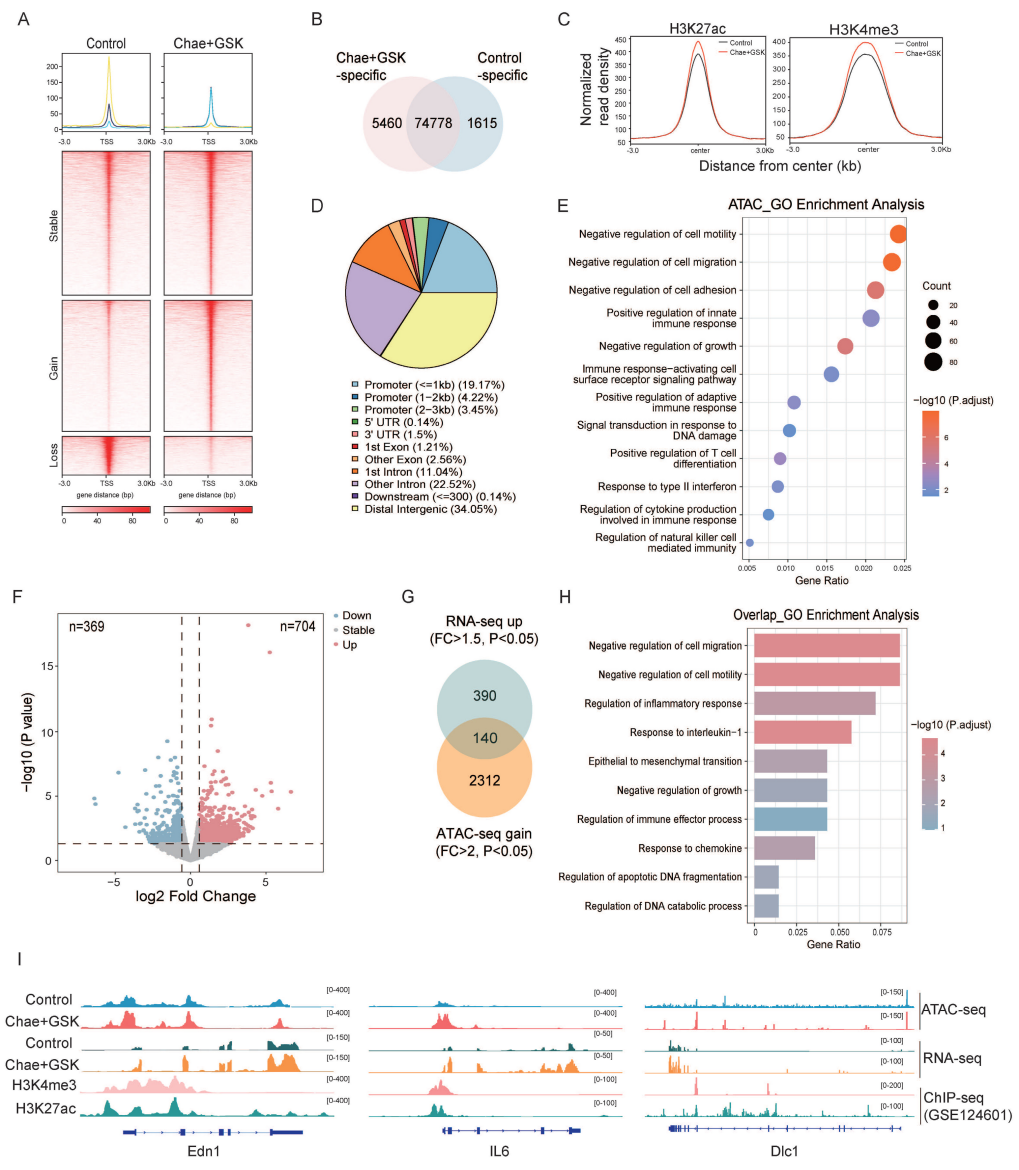
Multi-omics analysis reveals chromatin accessibility changes induced by dual inhibitors. (A) Heatmap and profile of ATAC-seq signals sorted on the basis of differential peaks in the dual-inhibitor group. (B) Total number of peaks identified by ATAC-seq analysis of control and dual-inhibitor groups. (C) Profile plot of ATAC-seq read density across H3K27ac and H3K4me3 ChIP-seq peak centers. (D) Pie chart showing the genomic distribution of treat-specific open chromatin regions. (E) GO analysis for genes corresponding to the up-regulated peaks in B. (F) Volcano plot showing downregulated and upregulated genes in dual-inhibitor treat versus control. (G) Venn diagram showing the intersection of genes with increased accessibility and upregulated genes at the transcriptional level. (H) GO analysis for the overlapped genes in G. (I) Genomic tracks of representative genes in ATAC-seq, RNA-seq, and ChIP-seq.

To elucidate the mechanism underlying dual inhibitor-mediated suppression of breast cancer progression, multi-omics analyses were performed on tumor tissues isolated from Py8119-inoculated C57BL/6 mice. First, chromatin accessibility changes induced by dual inhibitor treatment were assessed using assay for transposase-accessible chromatin with high-throughput sequencing (ATAC-seq). Dual inhibition of SUV39H1 and PRMT1 resulted in an increase in chromatin accessibility across multiple genomic regions, identified 5,460 gained and 1,615 lost peaks in dual inhibitor-treated samples compared to controls (Fig. 5A-B). The individual contributions of PRMT1 and SUV39H1 to the observed chromatin accessibility changes were exhibited on the basis of differential peaks in either PRMT1 or SUV39H1 inhibition alone (Supplementary Fig. 5A-B). We then compared our ATAC-seq data with publicly available ChIP-seq datasets for the active chromatin marks H3K27ac and H3K4me3 (GSE178144). The results showed substantial overlap between the open chromatin regions induced by dual inhibitor treatment and the peak regions of both H3K27ac and H3K4me3, suggesting their potential for transcriptional activation (Fig. 5C and Supplementary Fig. 5C-D). These open chromatin regions were predominantly located in promoter regions, intergenic regions and intronic regions (Fig. 5D). GSEA enrichment analysis indicated that many of the up-regulated peaks were associated with biological processes such as cell migration, growth, immune response, and DNA damage response (Fig. 5E).

Next, RNA-seq analysis identified 704 up-regulated and 369 down-regulated genes in dual inhibitor-treated samples compared to controls (Fig. 5F). The gained peaks from the ATAC-seq dataset were integrated with the up-regulated genes from the RNA-seq dataset, yielding 140 overlapping genes (Fig. 5G). These genes were enriched in pathways associated with the regulation of cell migration, growth, inflammatory, and immune response (Fig. 5H). Representative genes associated with favorable prognosis in breast cancer patients were selected to illustrate their genomic accessibility profiles (Fig. 5I and Supplementary Fig. 6). Collectively, these findings demonstrate that the increase in chromatin accessibility induced by dual inhibition of PRMT1 and SUV39H1 contributes mechanistically to the suppression of tumor progression.

### The clinical relevance of targeting PRMT1 and SUV39H1 in breast cancer

**Figure 6:**
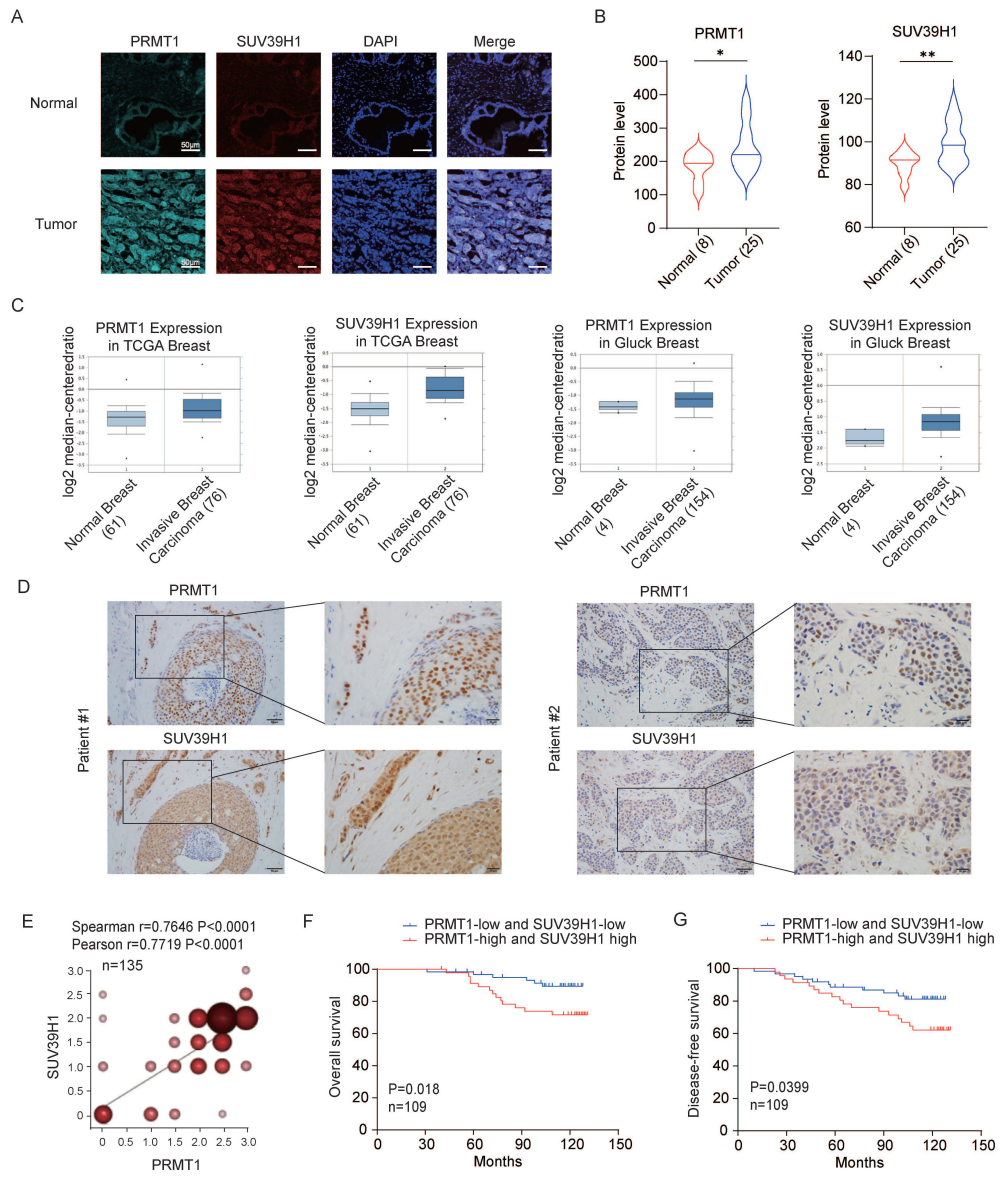
High PRMT1 and SUV39H1 is associated with poor outcomes in breast cancer patients. (A-B) Multiple IF staining was performed in tissue sections from 25 cases of breast invasive ductal carcinoma and 8 cases of normal breast tissue using PRMT1 and SUV39H1 antibodies. Representative images (A) and the fluorescence intensities of PRMT1 and SUV39H1 (B). *p < 0.05, **p < 0.01 by unpaired t test with Welch's correction. (C) Analysis of expression of SUV39H1 and PRMT1 in breast cancer patients' samples obtained from Oncomine datasets. (D-G) PRMT1 and SUV39H1 protein expression levels were assessed by immunohistochemistry (IHC) in a tissue microarray comprising 135 breast cancer patients. Representative IHC images illustrating high-expression (patient #1) and low-expression (patient #2) cases (D). Pearson and Spearman correlation tests (n=135) (E). Kaplan-Meier survival analysis was performed using overall survival data from breast cancer patients, based on immunohistochemical expression levels in tissue sections. Patients were stratified into a “both high-expression” group (n = 47) and a “both low-expression” group (n = 62) (F-G).

To investigate the clinical relevance of the epigenetic regulators PRMT1 and SUV39H1 in breast cancer, we first performed immunofluorescence staining to evaluate their expression in both breast cancer and normal tissues. Both proteins displayed significantly stronger signals in tumor tissues relative to normal tissues (Fig. 6 A-B), a result consistent with data from the TCGA database (Fig. 6C), thereby supporting their potential oncogenic functions in breast cancer development. Next, immunohistochemical analysis was carried out on human breast cancer tissue microarrays to assess protein expression levels. A positive correlation was observed between PRMT1 and SUV39H1 expression in breast tumor specimens (Fig. 6D-E). Survival analysis revealed that patients with co-high expression of PRMT1 and SUV39H1 exhibited significantly worse overall survival and disease-free survival compared to those with co-low expression (Fig. 6F-G). These findings collectively suggest that the concurrent overexpression of PRMT1 and SUV39H1 may serve as a prognostic indicator for poor outcomes in breast cancer patients.

**Figure 7:**
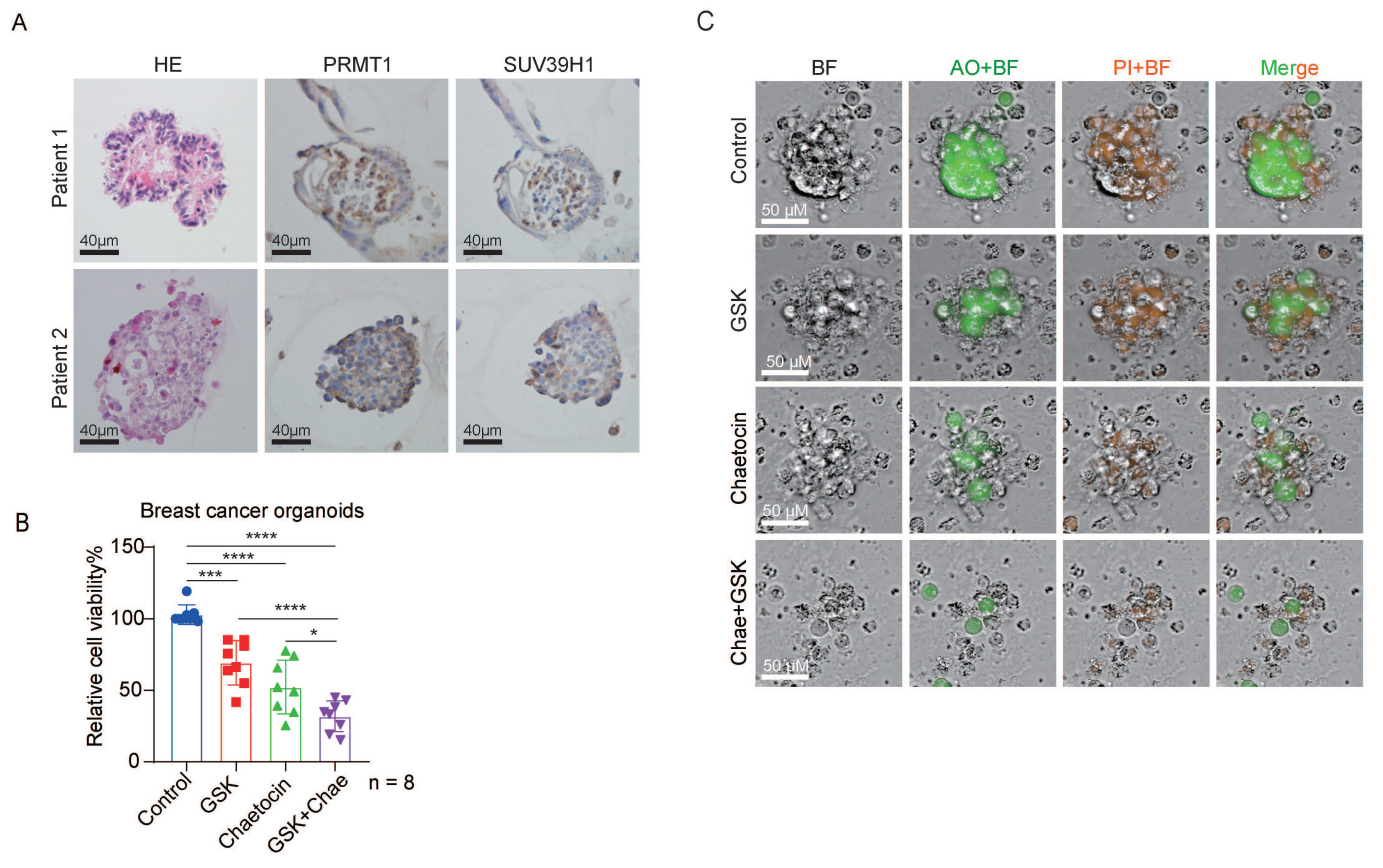
Dual inhibition suppresses the growth of breast cancer organoids. (A) Representative HE staining images, immunohistochemistry staining of PRMT1 and SUV39H1 in breast cancer organoids. (B) The viability of breast cancer organoids derived from 8 patients was assessed after 72-hour treatment with 0.1% DMSO, 200 nM GSK, 100 nM Chaetocin, 200 nM GSK plus 100 nM Chaetocin, using the CellTiter-Glo 3D Cell Viability Assay. Data are presented as mean ± SD. ***p < 0.001, and ****p < 0.0001 by one-way ANOVA (n=8). (C) Representative AO/PI staining images from B.

Furthermore, we examined the expression levels of PRMT1 and SUV39H1 in breast cancer organoids, confirming strong expression of both epigenetic regulators (Fig. 7A). Breast cancer organoids were treated with chaetocin alone, GSK3368715 alone, or the combination of both inhibitors. Consistent with our *in vivo* results, dual inhibition significantly suppressed organoid growth compared to single-agent treatments (Fig. 7B). Additionally, the combined treatment markedly reduced organoid size, as evidenced by live-cell imaging (Fig. 7C). Collectively, these results demonstrate that dual inhibition of PRMT1 and SUV39H1 impairs breast cancer progression.

## Discussion

In this study, we identify a promising therapeutic strategy for breast cancer via dual pharmacological inhibition of the epigenetic methyltransferases PRMT1 and SUV39H1. This dual inhibition synergistically suppresses breast cancer cell growth and metastasis by increasing the chromatin accessibility across multiple genomic regions. Furthermore, dual inhibition upregulates tumor cell-intrinsic PD-L1 expression, thereby sensitizing tumors to anti-PD-1 immunotherapy.

GSK3368715, a selective small-molecule inhibitor of PRMT1, exhibits potent antiproliferative activity across multiple cancer types in both cellular and murine models 9. This compound entered a phase I clinical trial for the treatment of solid tumors and diffuse large B-cell lymphoma [NCT03666988]. However, the trial was prematurely terminated due to limited clinical efficacy and severe adverse events—a higher-than-expected incidence of thromboembolic events at the 200 mg dose and an absence of measurable clinical benefit at lower doses (100 mg) 27. It underscores a narrow therapeutic window and highlight the critical need to optimize dosing strategies that preserve antitumor efficacy while reducing prothrombotic risk. To address this challenge, our study employed a rationally selected intermediate dose (150 mg) of GSK3368715 in combination with the SUV39H1 inhibitors (chaetocin and F5446). The dual inhibition of PRMT1 and SUV39H1 yields synergy, not merely additive effects (Supplementary Fig. 1G and Supplementary Fig. 3C). Given the thromboembolic incidence observed with monotherapy, we rigorously assessed the prothrombotic potential of the combination regimen through quantitative measurement of serum TXB2 levels and hematological indices. No significant hematologic toxicity was observed (Fig. 2I-L), and the regimen was well tolerated in murine breast cancer models. Thus, dual PRMT1-SUV39H1 inhibition at optimized doses represents a therapeutically viable and mechanistically rational strategy for breast cancer treatment.

Prior work from our group demonstrated that PRMT1 interacts with SUV39H1 and methylates it at R378, which blocks the binding of SUV39H1 and E3 ligase MDM2, resulting in the stabilization of SUV39H1 28. This establishes a modification-dependent regulatory axis between the two enzymes. Here, PRMT1 inhibition by GSK3368715 promotes SUV39H1 ubiquitination and subsequent proteasomal degradation (Fig. 1C-E). The rescue experiments using the degradation-resistant SUV39H1-K87A mutant provide direct functional evidence that the synergistic antitumor effect arises from coordinated disruption of the PRMT1-SUV39H1 regulatory axis, rather than independent parallel pathways.

Emerging evidence implicates the roles of PRMT1 and SUV39H1 in antitumor immunity. SUV39H1 inhibition can trigger both dsRNA and dsDNA sensing 20, while PRMT1 loss can activate either dsDNA- or dsRNA-specific responses depending on cellular context 29, 30. Here, dual inhibition of PRMT1 and SUV39H1 synergistically enhances the infiltration of both CD8+ T cells and NK cells. The increase in immune cell infiltration is predominantly driven by tumor cell-intrinsic epigenetic reprogramming, as confirmed by our *in vitro* assays, which showed potent induction of IFN-β and several key chemokines upon dual-inhibitor treatment of tumor cells. However, arginine methylation and H3K9 methylation is implicated in T cell activation and differentiation 31, 32. Thus, direct immune cell modulation by dual inhibitors cannot be excluded. The causal regulation of dual inhibition on CD8^+^ T cells and NK cells *in vivo* warrants further investigation.

Although PRMT1 and SUV39H1 inhibitors effectively activate the tumor immune microenvironment, the antitumor efficacy of dual PRMT1-SUV39H1 inhibition in the immunocompetent Py8119 xenograft model (Fig. 3B) was less pronounced than that observed in the immunocompromised MDA-MB-231 model (Fig. 2B). This discrepancy arises from two complementary mechanisms: first, MDA-MB-231 cells exhibit greater sensitivity to both chaetocin and GSK3368715 than Py8119 cells *in vitro* (Supplementary Fig. 1C-F); second, dual inhibition of PRMT1 and SUV39H1 enhances CD8^+^ T cell and NK cell infiltration, but concurrently induces marked PD-L1 upregulation in tumor cells (Fig. 3N), a canonical adaptive immune resistance mechanism. Thus, dual PRMT1-SUV39H1 inhibition exerts a dual-edged immunomodulatory effect: it augments cytotoxic lymphocyte infiltration while simultaneously engaging an adaptive immune escape pathway.

Given that prior studies have established a link between PRMT1 or SUV39H1 inhibition and PD-L1 expression 20, 29, we propose that dual inhibition of PRMT1 and SUV39H1 synergistically upregulates PD-L1 expression through two complementary epigenetic mechanisms. First, inhibition of SUV39H1 reduces H3K9me3 occupancy at the PD-L1 promoter. Second, PRMT1 inhibition promotes SUV39H1 degradation, thereby indirectly decreasing H3K9me3 abundance. Thus, concomitant PD-L1 blockade is necessary to fully realize the therapeutic potential of dual-inhibition.

## Supplementary Material

Supplementary figures.

## Data Availability

The raw sequence data reported in this paper have been deposited in the Genome Sequence Archive [33] in National Genomics Data Center [34], China National Center for Bioinformation / Beijing Institute of Genomics, Chinese Academy of Sciences (GSA: CRA036058) that are publicly accessible at https://ngdc.cncb.ac.cn/gsa.

## References

[1] Li WJ, He YH, Yang JJ, Hu GS, Lin YA, Ran T (2021). Profiling PRMT methylome reveals roles of hnRNPA1 arginine methylation in RNA splicing and cell growth. Nature communications.

[2] Poornima G, Shah S, Vignesh V, Parker R, Rajyaguru PI (2016). Arginine methylation promotes translation repression activity of eIF4G-binding protein, Scd6. Nucleic acids research.

[3] Vadnais C, Chen RY, Fraszczak J, Yu ZB, Boulais J, Pinder J (2018). GFI1 facilitates efficient DNA repair by regulating PRMT1 dependent methylation of MRE11 and 53BP1. Nature communications.

[4] Wang XY, Qiu T, Wu YY, Yang CY, Li Y, Du GS (2021). Arginine methyltransferase PRMT5 methylates and stabilizes KLF5 via decreasing its phosphorylation and ubiquitination to promote basal-like breast cancer. Cell death and differentiation.

[5] Wang K, Luo L, Fu SY, Wang M, Wang ZH, Dong LX (2023). PHGDH arginine methylation by PRMT1 promotes serine synthesis and represents a therapeutic vulnerability in hepatocellular carcinoma. Nature communications.

[6] Giuliani V, Miller MA, Liu CY, Hartono SR, Class CA, Bristow CA (2021). PRMT1-dependent regulation of RNA metabolism and DNA damage response sustains pancreatic ductal adenocarcinoma. Nature communications.

[7] Yang YZ, Bedford MT (2013). Protein arginine methyltransferases and cancer. Nature Reviews Cancer.

[8] Wu Q, Schapira M, Arrowsmith CH, Barsyte-Lovejoy D (2021). Protein arginine methylation: from enigmatic functions to therapeutic targeting. Nature Reviews Drug Discovery.

[9] Fedoriw A, Rajapurkar SR, O'Brien S, Gerhart SV, Mitchell LH, Adams ND (2019). Anti-tumor Activity of the Type I PRMT Inhibitor, GSK3368715, Synergizes with PRMT5 Inhibition through MTAP Loss. Cancer cell.

[10] Srour N, Villarreal OD, Hardikar S, Yu ZB, Preston S, Miller WH (2022). PRMT7 ablation stimulates anti-tumor immunity and sensitizes melanoma to immune checkpoint blockade. Cell Reports.

[11] Li Y, Dobrolecki LE, Sallas C, Zhang XD, Kerr TD, Bisht D (2023). PRMT blockade induces defective DNA replication stress response and with PARP inhibition. Cell Rep Med.

[12] Thiebaut C, Eve L, Poulard C, Le Romancer M (2021). Structure, Activity, and Function of PRMT1. Life-Basel.

[13] Li ZW, Wang DD, Lu J, Huang BQ, Wang YB, Dong MC (2020). Methylation of EZH2 by PRMT1 regulates its stability and promotes breast cancer metastasis. Cell death and differentiation.

[14] Li WJ, Chen YC, Lin YA, Zou YQ, Hu GS, Yang JJ (2025). Hypoxia-induced PRMT1 methylates HIF2b to promote breast tumorigenesis via enhancing glycolytic gene transcription. Cell Reports.

[15] Li WJ, Huang Y, Lin YA, Zhang BD, Li MY, Zou YQ (2023). Targeting PRMT1-mediated SRSF1 methylation to suppress oncogenic exon inclusion events and breast tumorigenesis. Cell Reports.

[16] Ku BM, Eisenbarth D, Baek S, Jeong TK, Kang JG, Hwang D (2024). PRMT1 promotes pancreatic cancer development and resistance to chemotherapy. Cell Rep Med.

[17] Padeken J, Methot SP, Gasser SM (2022). Establishment of H3K9-methylated heterochromatin and its functions in tissue differentiation and maintenance. Nat Rev Mol Cell Bio.

[18] Peters AH, O'Carroll D, Scherthan H, Mechtler K, Sauer S, Schofer C (2001). Loss of the Suv39h histone methyltransferases impairs mammalian heterochromatin and genome stability. Cell.

[19] Schotta G, Ebert A, Krauss V, Fischer A, Hoffmann J, Rea S (2002). Central role of SU(VAR)3-9 in histone H3-K9 methylation and heterochromatic gene silencing. Embo Journal.

[20] Shen JZ, Qiu ZX, Wu QL, Finlay D, Garcia G, Sun DH (2021). FBXO44 promotes DNA replication-coupled repetitive element silencing in cancer cells. Cell.

[21] Chu Y, Chen Y, Guo H, Li M, Wang B, Shi D (2020). SUV39H1 regulates the progression of MLL-AF9-induced acute myeloid leukemia. Oncogene.

[22] Wang JF, Yin XM, He W, Xue W, Zhang J, Huang YR (2021). SUV39H1 deficiency suppresses clear cell renal cell carcinoma growth by inducing ferroptosis. Acta Pharm Sin B.

[23] Zhang Y, Lao W, Yang K, Kong X, Li Y, Yu X (2023). SUV39H1 is a novel biomarker targeting oxidative phosphorylation in hepatitis B virus-associated hepatocellular carcinoma. BMC cancer.

[24] Zhou L, Chen Z, Zou Y, Zhang X, Wang Z, Zhu H ASB7 is a negative regulator of H3K9me3 homeostasis. Science. 2025: eadq7408.

[25] Fang L, Hao Y, Yu H, Gu X, Peng Q, Zhuo H (2023). Methionine restriction promotes cGAS activation and chromatin untethering through demethylation to enhance antitumor immunity. Cancer cell.

[26] Yu Y, Wu JZ, Wang YL, Zhao T, Ma B, Liu YQ (2012). Kindlin 2 forms a transcriptional complex with β-catenin and TCF4 to enhance Wnt signalling. EMBO reports.

[27] El-Khoueiry AB, Clarke J, Neff T, Crossman T, Ratia N, Rathi C (2023). Phase 1 study of GSK3368715, a type I PRMT inhibitor, in patients with advanced solid tumors. British journal of cancer.

[28] Zhao W, Wang G, Wang P, Ma B, Liu B, Fu YA (2026). Arginine methylation-dependent stabilization of SUV39H1 promotes breast cancer growth. Oncogene.

[29] Liu J, Bu X, Chu C, Dai XM, Asara JM, Sicinski P (2023). PRMT1 mediated methylation of cGAS suppresses anti-tumor immunity. Nature communications.

[30] Tao HR, Jin C, Zhou LY, Deng ZZ, Li X, Dang WZ (2024). PRMT1 Inhibition Activates the Interferon Pathway to Potentiate Antitumor Immunity and Enhance Checkpoint Blockade Efficacy in Melanoma. Cancer research.

[31] Geoghegan V, Guo AL, Trudgian D, Thomas B, Acuto O (2015). Comprehensive identification of arginine methylation in primary T cells reveals regulatory roles in cell signalling. Nature communications.

[32] Pace L, Goudot C, Zueva E, Gueguen P, Burgdorf N, Waterfall JJ (2018). The epigenetic control of stemness in CD8 T cell fate commitment. Science.

[33] Zhang SS, Chen X, Jin EH, Wang AK, Chen TT, Zhang XL (2025). The GSA Family in 2025: A Broadened Sharing Platform for Multi-omics and Multimodal Data. Genom Proteom Bioinf.

[34] Bao YM, Zhang Z, Zhao WM, Xiao JF, Song SH, He SM (2024). Database Resources of the National Genomics Data Center, China National Center for Bioinformation in 2025. Nucleic acids research.

