# Supplementary material for: Dual inhibition of PRMT1 and SUV39H1 suppresses breast cancer progression and enhances immunotherapy response
Source: Int J Biol Sci. 2026 Jun 4;22(11):6100–14. doi: 10.7150/ijbs.130955 (PMC13282782; doi:10.7150/ijbs.130955)
Supplement: Supplementary file 1 — Supplementary figures. [file ijbsv22p6100s1.pdf]

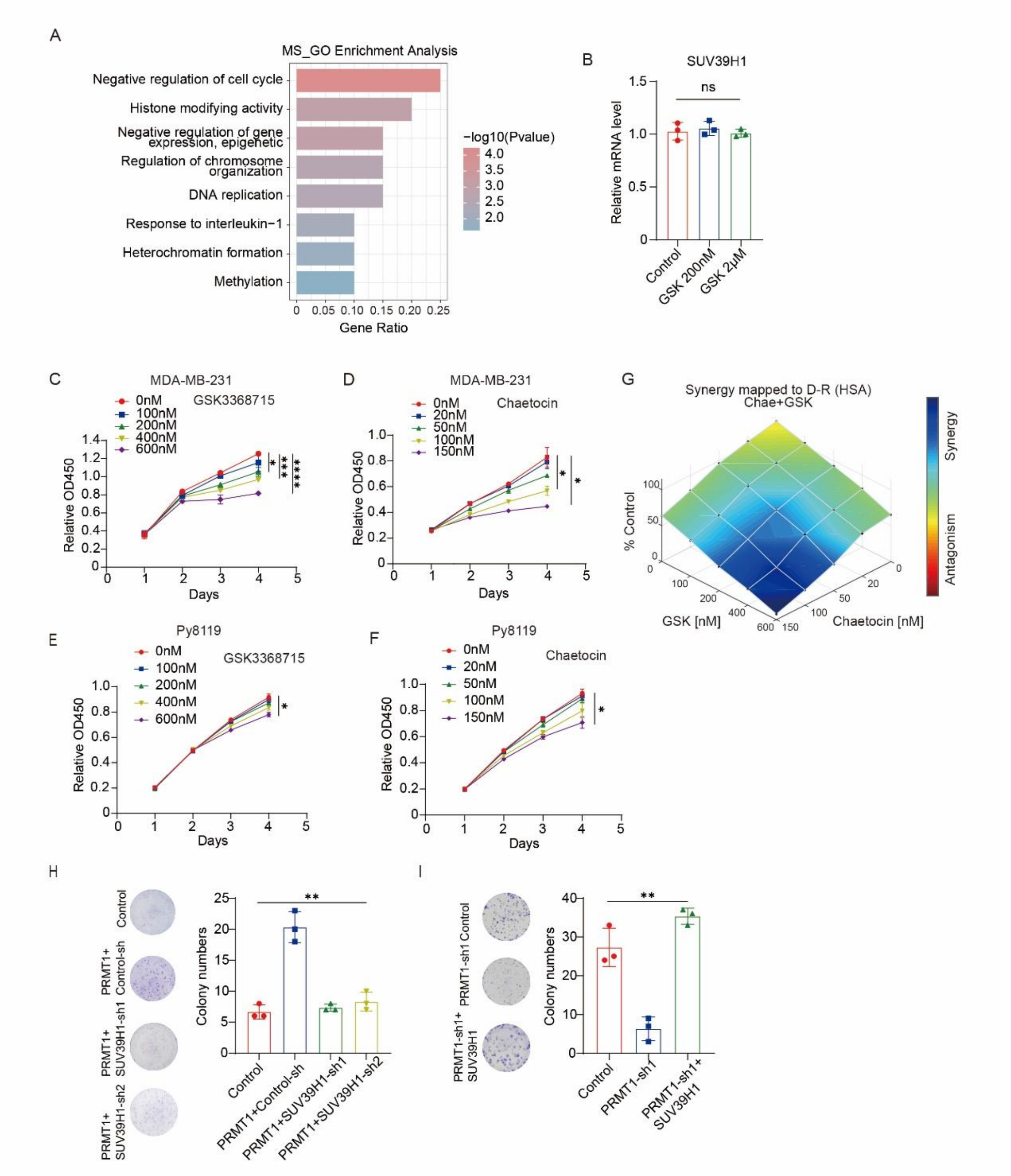

Fig. S1. Dual inhibition of PRMT1 and SUV39H1 suppresses breast cancer cell proliferation. (A) Total proteins were extracted from MDA-MB-231 cells treated with the PRMT1 inhibitor GSK3368715 (2 μM, 48 h) for LC-MS/MS. GO analysis for the differential proteins in GSK3368715-treated versus untreated cells ($P < 0.05$, fold change > 2.0). (B) MDA-MB-231 cells were treated with GSK3368715 (200nM/2 μM, 48 h), and total RNA was extracted for RT-PCR to detect the mRNA level of SUV39H1. (C-D) MDA-MB-231 cells were treated by GSK3368715 or chaetocin at different doses, and performed WST-1 cell proliferation assays. (E-F) Py8119 cells were treated by GSK3368715 or chaetocin at different doses, followed by WST-1 cell proliferation assays. *$p< 0.05$, ***$p< 0.001$, ****$p < 0.0001$ by two-way ANOVA with Geisser-Greenhouse correction. (G) Combenefit analysis was performed to quantitatively evaluate the drug interaction between GSK3368715 and chaetocin by HAS model. (H) Control-sh or SUV39H1-sh was

transfected into PRMT1-overexpressing MDA-MB-231 cells, followed by a colony formation assay. (I) Flag-SUV39H1 was transfected into PRMT1-shRNA treated MDA-MB-231 cells, followed by a colony formation assay. Data represent the mean ± SD. **p < 0.01 by One-way ANONA.

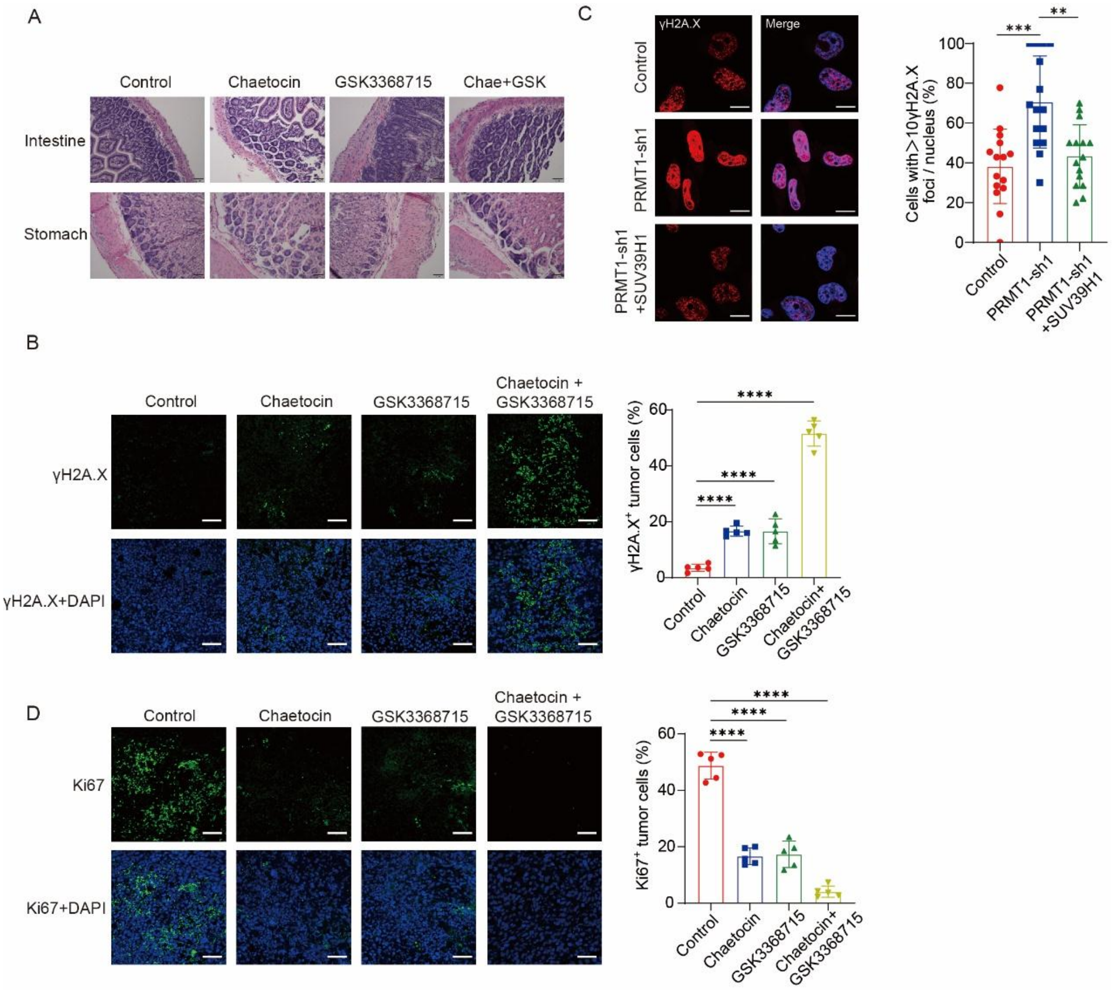

Fig. S2. Combination therapy of PRMT1 and SUV39H1 inhibitors suppresses breast cancer growth. (A) H&E staining of intestine and stomach tissues from mice treated with GSK3368715, chaetocin, or their combination. (B) The tumor tissues of 4 groups were made into paraffin-embedded sections, and stained by anti-γH2A.X antibody. Tumor cells of γH2A.X positive were statistically analyzed. (C) Immunofluorescence of γH2A.X was measured to assess the formation of γH2A.X foci in indicated stable cells. Representative images and statistical analysis are shown. (D) The tumor tissues of 4 groups were stained by anti-Ki67 antibody. Tumor cells of Ki67 positive were statistically analyzed.

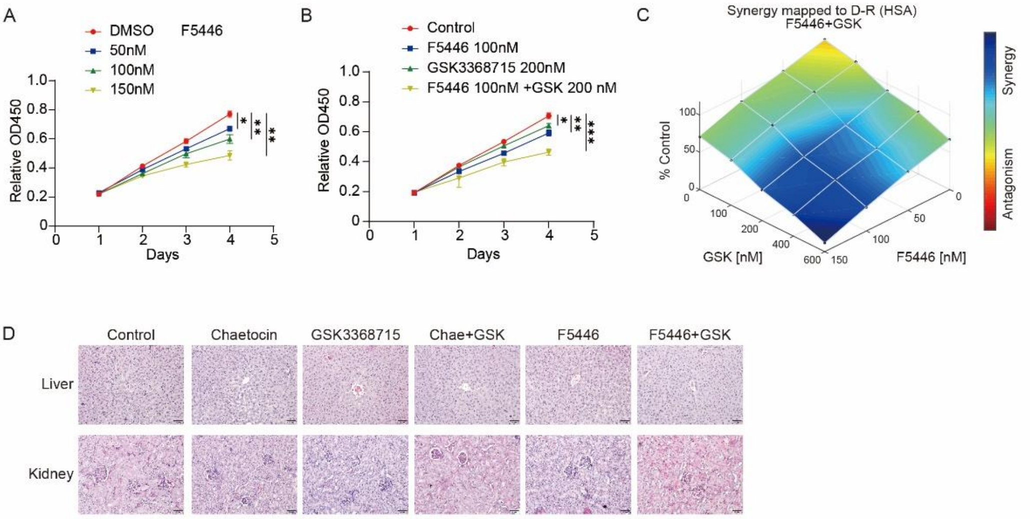

Fig. S3. SUV39H1 inhibitor F5446 exhibits significant synergy with GSK3368715 to inhibit cell proliferation. (A) MDA-MB-231 cells were treated by F5446 at different doses, and performed WST-1 cell proliferation assays. (B) MDA-MB-231 cells were treated by GSK3368715 (200 nM) and/or F5446 (100 nM) followed by WST-1 assays. *p< 0.05, **p< 0.01 by two-way ANOVA with Geisser-Greenhouse correction. (C) Combenefit analysis was performed to quantitatively evaluate the drug interaction between GSK3368715 and F5446 by HAS model. (D) H&E staining of the liver and kidney tissues from the mice in Fig. 3B.

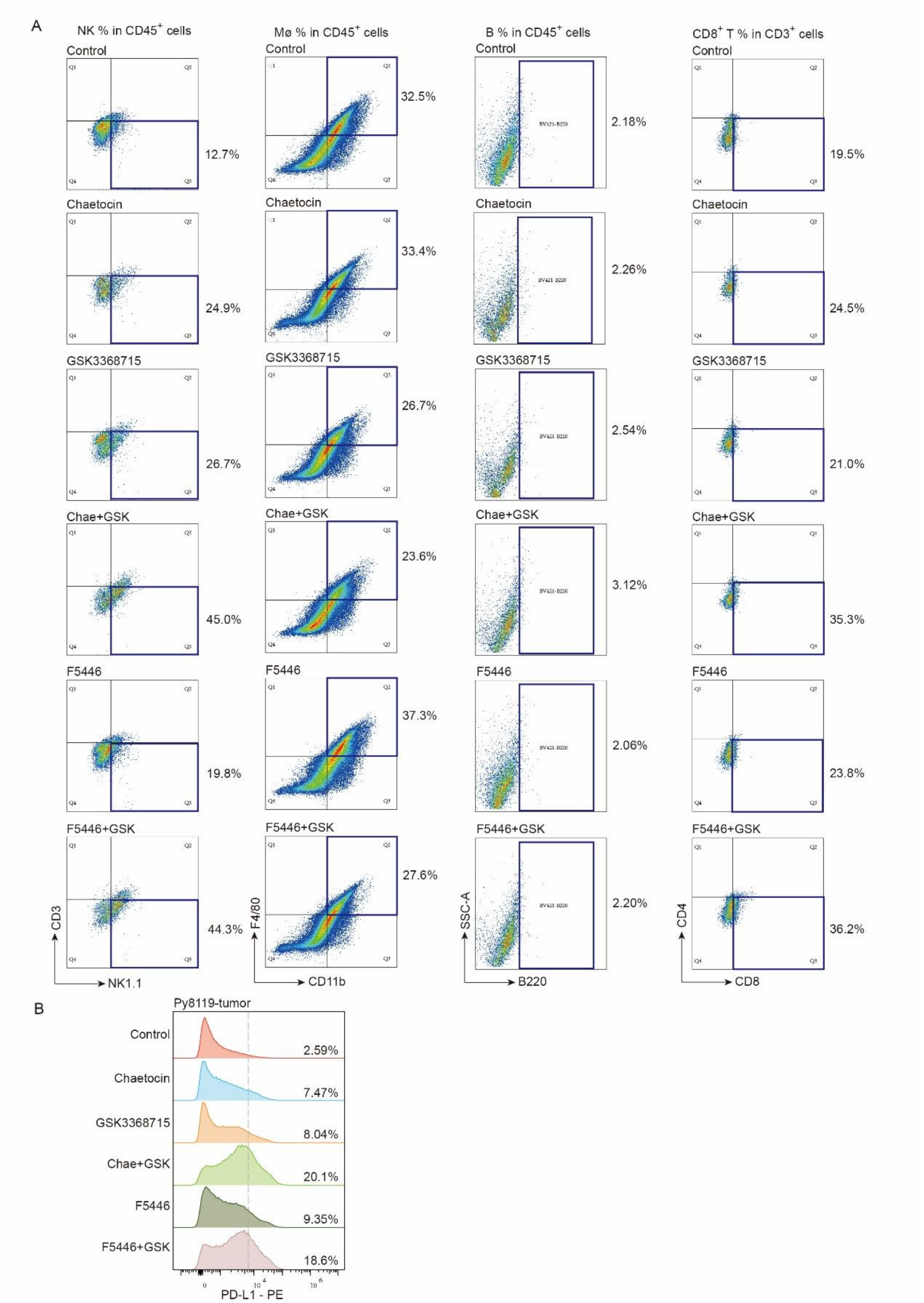

Fig. S4. Tumors from six groups were digested into a single cell suspension for multiparametric flow cytometry. Cell populations of CD8$^+$ T cells, NK cells, macrophages, B cells (A), and PD-L1$^+$ cells (B) were quantified.

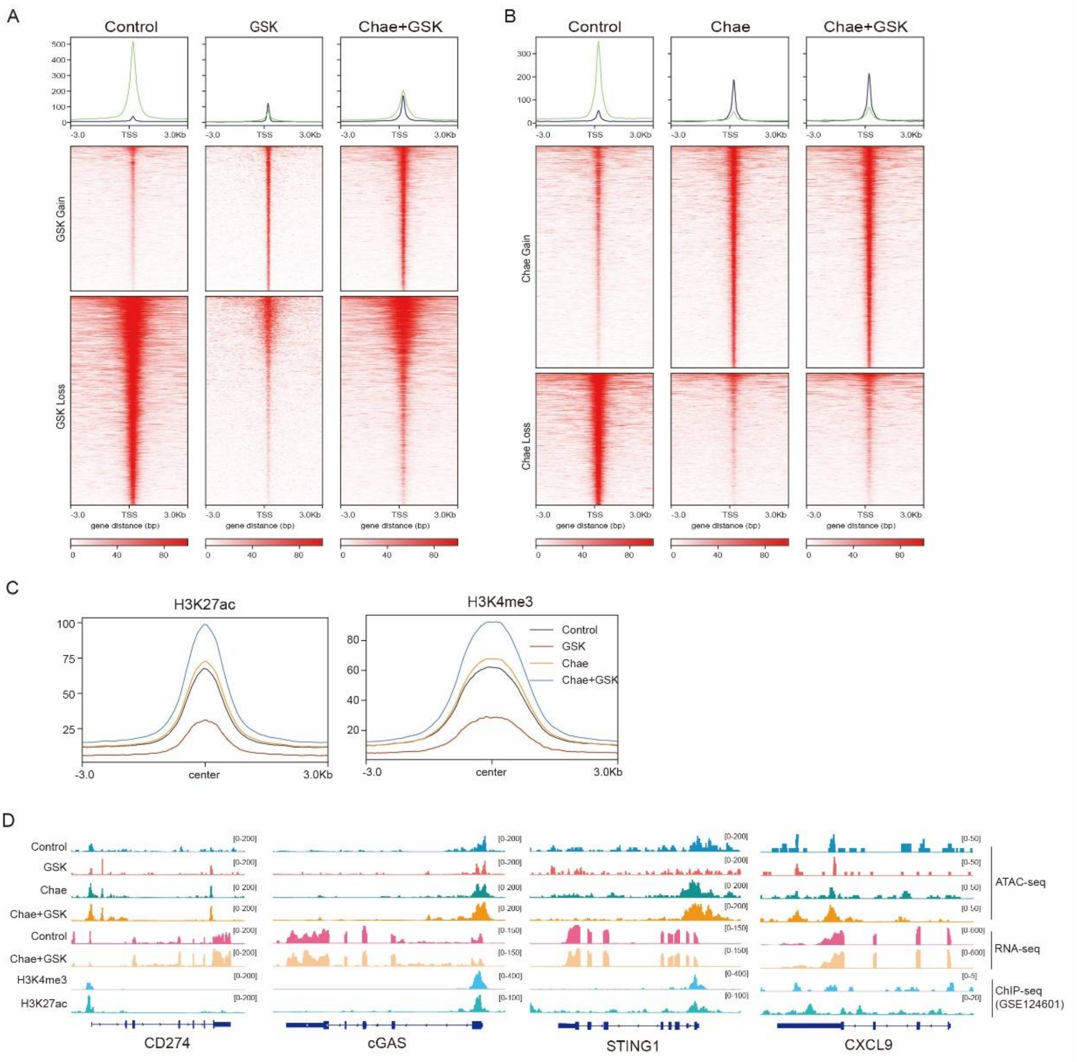

Fig. S5. Multi-omics analysis reveals chromatin accessibility changes induced by dual inhibitors. (A) Heatmap and profile of ATAC-seq signals sorted based on differential peaks identified in monotherapy with GSK3368715. (B) Heatmap and profile of ATAC-seq signals sorted based on differential peaks identified in monotherapy with chaetocin. (C) Profile plot of ATAC-seq read density across H3K27ac and H3K4me3 ChIP-seq peak centers. (D) Genomic tracks of representative genes in ATAC-seq, RNA-seq, and ChIP-seq.

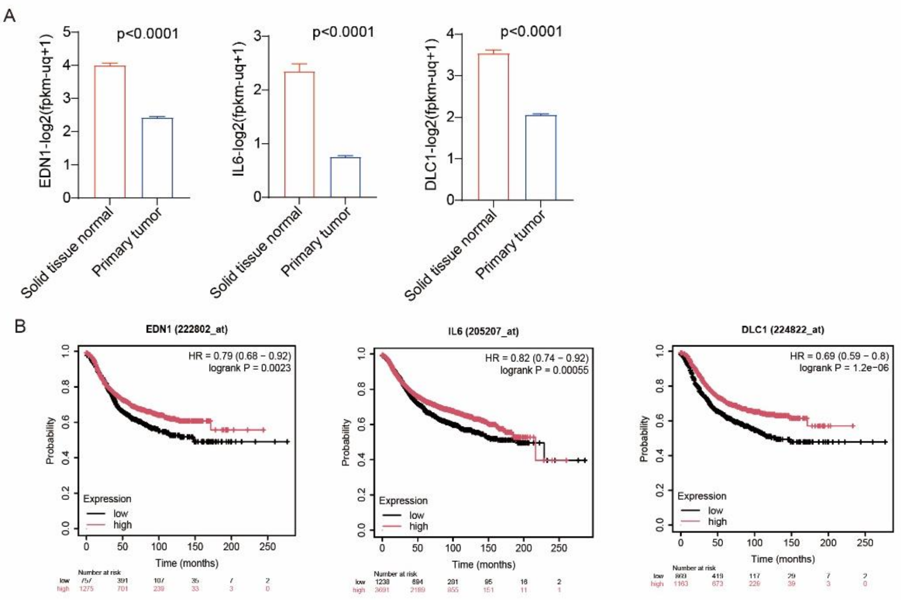

Fig. S6. Three representative genes regulated by combination therapy are associated with favorable outcomes in breast cancer patients. (A) Analysis of expression of EDN-1, IL-6, and Dlc1 in breast cancer patients' samples obtained from Oncomine datasets. (B) Kaplan-Meier analysis of overall survival according to mRNA levels of EDN1, IL-6, and DLC1 in breast cancer patients using the Kaplan-Meier Plotter database.
